# Brief communication: β-cell function influences dopamine receptor availability

**DOI:** 10.1371/journal.pone.0212738

**Published:** 2019-03-08

**Authors:** Julia P. Dunn, Naji N. Abumrad, Bruce W. Patterson, Robert M. Kessler, Robyn A. Tamboli

**Affiliations:** 1 Department of Medicine, Vanderbilt University School of Medicine, Nashville, Tennessee, United States of America; 2 Department of Medicine, Washington University School of Medicine, St. Louis, Missouri, United States of America; 3 Veterans Administration St. Louis Health Care System, St. Louis, Missouri, United States of America; 4 Department of Surgery, Vanderbilt University School of Medicine, Nashville, Tennessee, United States of America; 5 Department of Radiology, Vanderbilt University School of Medicine, Nashville, Tennessee, United States of America; George Washington University School of Medicine and Health Sciences, UNITED STATES

## Abstract

We aim to identify physiologic regulators of dopamine (DA) signaling in obesity but previously did not find a compelling relationship with insulin sensitivity measured by oral-minimal model (OMM) and DA subtype 2 and 3 receptor (D2/3R) binding potential (BP_ND_). Reduced disposition index (DI), a β-cell function metric that can also be calculated by OMM, was shown to predict a negative reward behavior that occurs in states of lower endogenous DA. We hypothesized that reduced DI would occur with higher D2/3R BP_ND_, reflecting lower endogenous DA. Participants completed PET scanning, with a displaceable radioligand to measure D2/3R BP_ND_, and a 5-hour oral glucose tolerance test to measure DI by OMM. We studied 26 age-similar females without (n = 8) and with obesity (n = 18) (22 vs 39 kg/m^2^). Reduced DI predicted increased striatal D2/3R BP_ND_ independent of BMI. By accounting for β-cell function, we were able to determine that the state of insulin and glucose metabolism is pertinent to striatal D2/3R BP_ND_ in obesity.

**Clinical Trial Registration Number**: NCT00802204

## Introduction

Diminished dopamine (DA) signaling and food reward are associated with obesity and are postulated to contribute to and/or perpetuate obesity. The mechanisms of the anorexic effects of insulin include regulating food reward through DA signaling, and thus, impaired insulin sensitivity (i.e. insulin resistance) is expected to dysregulate DA signaling [1] as occurs in rodent models of diet induced obesity (DIO) [2]. We previously reported that higher body mass index (BMI) and lower fasting acyl ghrelin concentrations were associated with increased striatal DA subtype 2 and 3 receptor (D2/3R) binding potential (BP_ND_), which we interpreted to reflect lower levels of endogenous DA competing with the displaceable radioligand, [^18^F]fallypride [3]. Reduced striatal DA levels occur in DIO rodents [4] and one human report had trend level data demonstrating reduced pharmacologically-induced DA release in obesity [5]. Using the same radioligand we utilized, [^18^F]fallypride, others found positive relationships between BMI and D2/3R BP_ND_ in the dorsal striatum (caudate and putamen) with conflicting findings in the ventral striatum [6, 7]. In our previous report, insulin resistance also predicted higher striatal D2/3R binding, (i.e. lower endogenous DA), but this effect was not independent of BMI [3]. Eisenstein et al used β-cell function and [^11^C](N-methyl)benperidol([^11^C]NMB), a non-displaceable, D2 receptor-selective radioligand, to examine the relationship between DA signaling, obesity, and insulin. They did not find associations between striatal D2R levels and BMI or β-cell function (determined by disposition index, DI). This lack of relationship between BMI and receptor levels with a non-displaceable radioligand supported our interpretation that differences in endogenous DA levels were a predominate factor defining the relationship we identified with BMI and receptor levels measured with a displaceable radioligand. Eisenstein et al did report that β-cell function was associated with increased delayed discounting, a detrimental reward behavior which reflects impaired inhibitory control and is attenuated by agents that increase extracellular DA levels. Essentially, they found that impaired β-cell function occurred with a behavior that is present in a state of decreased endogenous DA [8]. This finding prompted us to re-examine our data to determine the relationship of β-cell function measured by DI to striatal D2/3R BP_ND_ estimated with a displaceable radioligand. Further we sought to determine if any identified relationships were independent of BMI as our primary aim is to define physiologic regulators of DA signaling.

## Methods

The study protocol was approved by the Vanderbilt University Institutional Review Board and all participants gave written informed consent. We studied 26 weight-stable females, 8 non-obese (22±3 kg/m^2^) and 18 obese (39±6 kg/m^2^) of similar age (Table 1), 22 who were included in our prior report [3]. Screening included history and physical exam, laboratory testing including urine drug screen, and magnetic resonance imaging (MRI) of the brain. Exclusion included pregnancy, significant current psychiatric, neurologic or medical condition. One participant had diet-controlled type 2 diabetes mellitus. Individuals were also excluded if current tobacco use, substance abuse or heavy alcohol use, or if treated with central acting medications or insulin sensitizing agents in the preceding six months.

**Table 1:** Total cohort of participants that completed baseline PET imaging and OGTT.

	Non-obese (n = 8)	Obese (n = 18)	p-value
**Weight (kg)**	59±7	106±17	
**BMI (kg/m^2^)**	22±3	39±6	
**Age (y)**	41±9	39±8	0.489
**SI** (10^−4^ * min^-1^ *μU^-1^ * mL)	11.2±4.1	3.9±2.5	<0.001
**ϕ_total_** (10^9^ min-1)	26.7±8.6	30.8±10.3	0.336
**DI** (10^6^ min^-2^ *μU^-1^ * mL)	29.6±13.7	10.8±6.67	0.005
**Regional D2/3R BP_ND_**			
**Caudate**	28.9±3.3	32.6±2.8	0.006
**Putamen**	34.2±3.8	37.6±2.5	0.013
**Ventral Striatum**	19.1±3.8	22.1±2.6	0.030

As detailed previously [3], before admission to the Vanderbilt University Clinical Research Center (CRC) participants were requested to refrain from exercise, alcohol and excess caffeine for 48 hours. On the day of admission, at ~18:30h after an eight-hour fast, blood was collected (with serine protease inhibitor and subsequent plasma acidification for acyl ghrelin measurement) then positron emission tomography (PET) scanning with [^18^F]fallypride was completed. Participants stayed overnight at the CRC and the next morning underwent a five-hour 75 gram oral glucose tolerance test (OGTT) with 11 blood draws for glucose, insulin and C-peptide measurement [9]. The oral-minimal model (OMM) was applied to provide estimates of insulin sensitivity (SI) and insulin secretion (ϕ_total_) by modeling the relationships of glucose with insulin and C-peptide with glucose, respectively [9]. The disposition index (DI) is the product of SI and ϕɸ_total_ and describes the β-cells’ ability to respond to a decrease in insulin sensitivity by appropriately increasing insulin secretion [10].

Imaging techniques and analysis were also completed as previously detailed [3]. Briefly, T1 weighted images of the brain were obtained on either a 1.5T or 3T MRI at screening. PET scanning was initiated with a bolus injection of [^18^F]fallypride and was completed in three scan sequences over approximately 3.5 hours. For imaging analysis, the serial PET scans were co-registered to each other and to the thin section T1-weighted MRI scans using a mutual information rigid body algorithm. The full reference region method was used to calculate regional DA D2/D3R BP_ND_ with the cerebellum as the reference region. Region of interest (ROI) analysis of the caudate, putamen, and ventral striatum was used due to the regions’ relevance to reward behaviors and being the most consistently reported ROIs in the literature. The caudate and putamen were manually drawn on axial slices of the MRI at approximately 2–12 mm above the anterior commissure—posterior commissure line. The ventral striatum was delineated on coronal slices using established criteria [11]. The ROIs were delineated bilaterally on the MRI images and transferred to the coregistered PET scans. The average value between the two hemispheres for each region was used for analysis.

Data are presented as mean ± standard deviation (SD) and non-obese vs obese, respectively. Demographic and outcome measures were compared using student t-tests. The relationship between striatal D2/3R BP_ND_ and age, BMI, SI, DI, ϕ_total_ were evaluated by linear regression. The β’s reported are the unstandardized coefficients.

## Results

**Fig 1:**
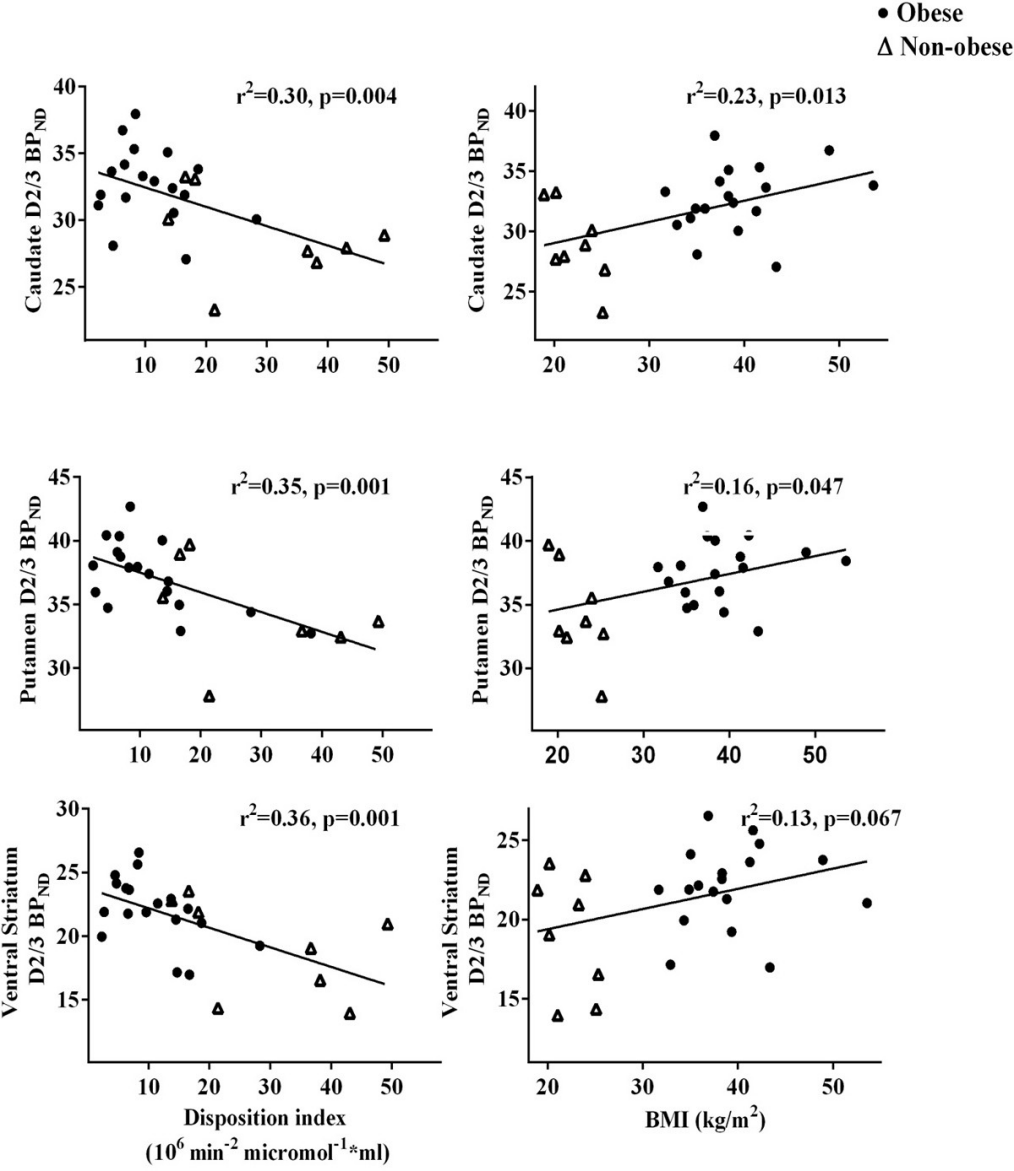
Linear regressions for striatal D2/3R availability with disposition index and D2/3R availability with BMI.

D2/3R BP_ND_ was 10–15% higher in the obese (caudate 29 vs. 33, p = 0.006; putamen 34 vs. 38, p = 0.013; ventral striatum 19 vs. 22, p = 0.030) (Table 1). We again found that higher BMI, lower insulin sensitivity (SI), and lower fasting acyl ghrelin levels predicted increased D2/3R binding throughout the striatum (Table 2, Fig 1). DI also had significant negative relationships with striatal D2/3R BP_ND_. Neither age (p-values = 0.11–0.60) nor ϕ _total_ (p-values = 0.42–0.98) were associated with regional receptor binding in our cohort_._ When adjusted for BMI, SI did not maintain relationships with D2/3R BP_ND_ (p-values = 0.1–0.2) but DI maintained robust negative relationships with D2/3R BP_ND_ (Table 3). DI explained all variance in D2/3R BP_ND_ attributed to BMI without significantly modifying parameter estimates for DI. Since in our previous report fasting acyl ghrelin concentrations were associated with striatal D2/3R BP_ND_ independent of BMI, we explored the relationship of both DI and fasting acyl ghrelin concentrations to receptor binding. When both were included as covariates to explain striatal D2/3R BP_ND_, DI continued to explain the majority of the variance (p values = 0.088–0.028) while acyl ghrelin concentrations did not maintain independent (p values≥0.15) relationships with striatal D2/3R BP_ND_; however, the parameter estimates for DI changed by 19–31%, demonstrating acyl ghrelin concentrations as a relevant modifier (Table 3).

**Table 2:** Univariate metabolic predictors of striatal D2/3R binding potential.

	BMI			SI			Acyl ghrelin			DI		
**Region**												
	R	β	p-value	R	β	p-value	R	β	p-value	R	β	p-value
**Caudate**	0.482	0.18	0.013	0.485	-0.39	0.012	0.511	-0.016	0.008	0.545	-0.14	0.004
**Putamen**	0.394	0.14	0.047	0.492	-0.36	0.011	0.549	-0.017	0.004	0.595	-0.15	0.001
**Ventral Striatum**	0.364	0.13	0.067	0.477	-0.34	0.014	0.491	-0.015	0.011	0.601	-0.15	0.001

**Table 3:** Multivariate relationships for striatal D2/3R binding potential with either DI and BMI or DI and fasting acyl ghrelin concentrations.

	Caudate			Putamen			Ventral Striatum		
	R	β	p-value	R	β	p-value	R	β	p-value
**DI**	0.589	-0.11	0.057	0.601	-0.14	0.012	0.602	-0.15	0.008
**BMI**		0.10	0.201		0.04	0.618		0.02	0.788
**DI**	0.592	-0.10	0.088	0.642	-0.11	0.049	0.623	-0.12	0.028
**Acyl ghrelin**		-0.009	0.184		-0.009	0.145		-0.006	0.323

## Discussion

**Fig 2:**
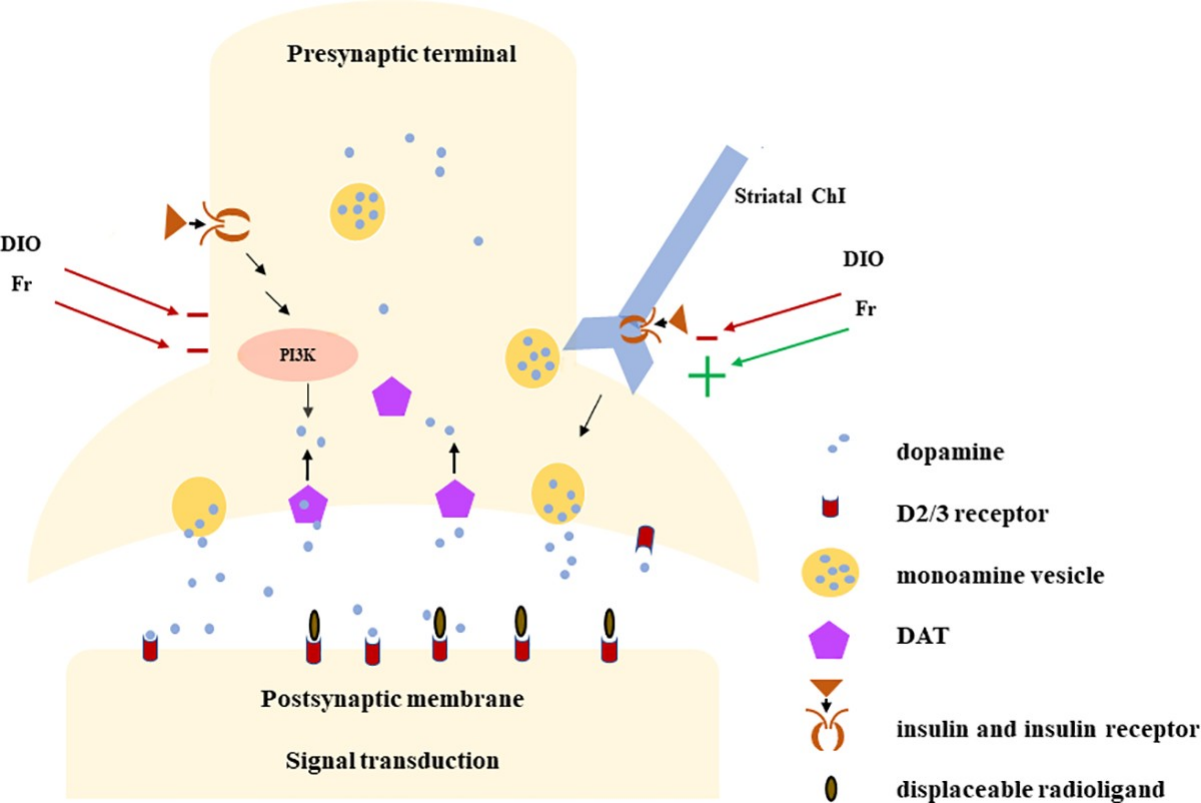
Presynaptic terminal: Simplified schema of insulin effects on DA neuron synapse in the striatum. Evidence supports cell surface DAT and striatal ChI excitability vary by diet-induced effects on both insulin secretion/levels and insulin sensitivity. Postsynaptic membrane: with PET imaging the displaceable radioligand competes with extracellular DA for binding.

We determined that impaired β-cell function (lower DI) is associated with higher striatal D2/3R binding. Again, this is interpreted as lower endogenous DA as we used a displaceable radioligand (Fig 2). DI allows insulin secretion to be interpreted in light of the prevailing insulin sensitivity, providing a quantitative index that estimates an individual’s state of glucose tolerance based on a physiologic challenge [12]. Our regressions demonstrate that this physiologic measure can describe over twice the variance in D2/3R binding as the non-physiologic BMI measure. The effect size we detail for DI with D2/3R binding is substantially greater than what others have reported for BMI with D2/3R binding using the same radioligand [7]. We did not find age to be a modifier of striatal D2/3R binding as others have reported; however it is important to note the DI declines with aging and can be modified by weight loss [13]. BMI is commonly used to explore the effect of obesity on central reward signaling, but the current work demonstrates its limitations. The state of insulin and glucose metabolism are pertinent to design and interpretation of studies of DA signaling in obesity. Further, if aspects of insulin and glucose metabolism are influencing central reward signaling, this presents the potential for personalized therapeutic targets in diseases of reward signaling and obesity.

Besides peripherally secreted insulin directly modifying DA signaling there is evidence for a dopaminergic gut-brain axis at least partially mediated via the vagus. There are hypothalamic-vagal-pancreatic connections with dopaminergic innervations[14] and peripheral insulin also modifies vagal afferents[15]. Intact vagal transmission is necessary for meal-induced increases in striatal DA release[16], however it is unknown if this is modified by insulin signaling. Parkinson’s Disease, a state of central dopaminergic depletion, occurs with impaired vagal activity and altered glucose-stimulated insulin secretion (GSIS) [17]. In rodents, depleting nigrostriatal DA alters colonic DA, D2R and DA type 1 receptor levels which is hypothesized to contribute to the gastrointestinal symptoms of Parkinson’s Disease [18]. We are not aware of any direct evidence that central DA depletion influences pancreatic β-cell DA signaling however, insulin sensitivity increases with acute deep brain stimulation (DBS) of the internal capsule, (which is known to increase striatal DA release) and decreases with acute pharmacologic depletion of extracellular DA [19]. At the level of the DA neuron (Fig 2), insulin increases cell-surface expression of the DA transporter (DAT) which takes DA back up into the presynaptic neuron. This function is impaired in insulin-deficient streptozotocin (STZ) diabetic rodents[20] and in insulin resistant (impaired insulin signaling) diet induced obese (DIO)[15] as both have reduced cell-surface DAT. DAT activity is also reduced in food restricted (Fr) animals[21] that have low insulin levels. While direct insulin exposure in the ventral tegmental area (VTA) causes reduced extracellular DA levels via increased DAT activity [22], Stouffer et al reported that direct insulin exposure in the striatum has competing effects on extracellular DA. In the striatum, insulin does promote DA reuptake via DAT but it also increases the excitability of striatal cholingergic interneurons (ChI) causing release of DA which is the predominant effect. As this is mediated by insulin receptors on ChI, it is modified by insulin sensitivity such that insulin resistant DIO obese and insulin sensitive food-restricted rodents have attenuated and amplified DA release, respectively[21]. This demonstrates that both insulin secretion/levels and insulin sensitivity influence DA signal transduction consistent with our current report.

We hypothesize that at least some of the inconsistencies in the literature related to DA signaling in obesity (reviewed by Dang, L et al) [7] are due to metabolic variation within obese cohorts. Other specifics of our protocol including weight stability, restraining from exercise [23] before imaging, fasting pre-scan [24], and evening scan times [25] are also posited to contribute to our findings as pre-clinical studies support these as relevant modifiers of DA signaling. The modification of the relationship between DI and D2/3R binding by ghrelin support both the relevance of fasting, when ghrelin reaches its peak, and the known effects of ghrelin on DA mediated reward signaling [1]. A more comprehensive understanding as to how weight gain modifies the dopaminergic gut/pancreatic-brain axis will be relevant to various disease processes including dysregulated reward signaling in obesity.

## Supporting information

S1 File (DOCX)
