# Supplementary material for: Brief communication: β-cell function influences dopamine receptor availability
Source: PLoS One. 2019 Mar 8;14(3):e0212738. doi: 10.1371/journal.pone.0212738 (PMC6407783; doi:10.1371/journal.pone.0212738)
Supplement: S1 File — (DOCX) [file pone.0212738.s001.docx]

| id | age | weight | bmi | ins_sens | di_total | phi_total | a_ghr_scan | d2_cau | d2_put | d2_vs |
| --- | --- | --- | --- | --- | --- | --- | --- | --- | --- | --- |
| 8 | 57 | 103.89 | 35.04 | 2.15 | 4.7 | 21.83 | 134.4 | 28.09 | 34.72 | 24.13 |
| 9 | 35 | 113.1 | 41.59 | 2.54 | 8.2 | 32.17 | 67.29 | 35.32 | 37.89 | 25.63 |
| 12 | 33 | 104.3 | 41.26 | 2.29 | 6.8 | 29.86 | 66.83 | 31.69 | 38.75 | 23.64 |
| 13 | 35 | 79.7 | 34.86 | 6.31 | 2.7 | 4.22 | 172.85 | 31.9 | 35.95 | 21.9 |
| 15 | 50 | 117.85 | 42.26 | 1.81 | 4.5 | 24.81 | 47.87 | 33.65 | 40.43 | 24.79 |
| 16 | 40 | 134.81 | 48.92 | 1.49 | 6.3 | 42.41 | 60.06 | 36.73 | 39.1 | 23.76 |
| 17 | 39 | 96.05 | 38.33 | 7.71 | 13.7 | 17.82 | 134.35 | 35.09 | 40.03 | 22.93 |
| 19 | 29 | 106.86 | 38.32 | 3.57 | 11.5 | 32.32 | 47.4 | 32.92 | 37.39 | 22.57 |
| 20 | 31 | 93.28 | 35.85 | 8.62 | 16.5 | 19.18 | 64.39 | 31.89 | 34.96 | 22.15 |
| 21 | 46 | 139.21 | 53.57 | 4.52 | 18.7 | 41.32 | 115.8 | 33.81 | 38.42 | 21.04 |
| 22 | 38 | 63.9 | 21.06 | 10.6 | 43.1 | 40.66 | 402.3 | 27.94 | 32.44 | 13.97 |
| 23 | 49 | 58.47 | 25.11 | 6.93 | 21.4 | 30.82 | 345.75 | 23.3 | 27.81 | 14.37 |
| 24 | 53 | 65.41 | 23.97 | 5.76 | 13.8 | 24.01 | 191.09 | 30.1 | 35.55 | 22.81 |
| 27 | 51 | 68.37 | 25.33 | 18 | 38.2 | 21.2 | 264.18 | 26.84 | 32.72 | 16.56 |
| 29 | 36 | 50.86 | 20.17 | 13.2 | 36.7 | 27.78 | 291.76 | 27.71 | 32.94 | 19.06 |
| 33 | 29 | 51.99 | 18.94 | 12.7 | 18.2 | 14.29 | 247.85 | 33.07 | 39.72 | 21.88 |
| 34 | 37 | 51.67 | 20.18 | 8.3 | 16.6 | 20.01 | 135.77 | 33.25 | 38.95 | 23.55 |
| 36 | 37 | 64.75 | 23.27 | 14.2 | 49.3 | 34.73 | 148.55 | 28.89 | 33.69 | 20.97 |
| 37 | 38 | 100.69 | 36.89 | 2.43 | 8.4 | 34.62 | 136.51 | 37.95 | 42.68 | 26.56 |
| 43 | 41 | 106.52 | 38.84 | 2.99 | 14.5 | 48.51 | 193.65 | 32.39 | 36.03 | 21.3 |
| 46 | 45 | 106.48 | 39.35 | 8.87 | 28.3 | 31.9 | 53.73 | 30.06 | 34.4 | 19.24 |
| 47 | 44 | 90.07 | 32.92 | 4.47 | 14.7 | 32.87 | 26.27 | 30.54 | 36.8 | 17.16 |
| 48 | 43 | 98.51 | 34.33 | 0.59 | 2.3 | 38.91 | 15.26 | 31.11 | 38.06 | 19.96 |
| 51 | 29 | 127.2 | 43.35 | 5.29 | 16.7 | 31.59 | 34.37 | 27.07 | 32.91 | 16.98 |
| 54 | 37 | 78 | 31.68 | 2.87 | 9.6 | 33.6 | 17.99 | 33.29 | 37.95 | 21.89 |
| 55 | 27 | 103.08 | 37.41 | 1.81 | 6.6 | 36.36 | 21.37 | 34.16 | 40.36 | 21.76 |

S1. Supporting information

Subject identifier id, Body Mass Index bmi, insulin sensitivity ins_sens, disposition index total di_total, insulin secretion (Φ_total)_ phi_total, acyl ghrelin at time of scan a_ghr_scan, dopamine 2/3 receptor availability d2, cau caudate, put putamen, vs ventral striatum
